# Integrating Videoconferencing Therapist Guidance Into Stepped Care Internet-Delivered Cognitive Behavioral Therapy for Child and Adolescent Anxiety: Noninferiority Randomized Controlled Trial

**DOI:** 10.2196/57405

**Published:** 2025-01-22

**Authors:** Sonja March, Susan H Spence, Larry Myers, Martelle Ford, Genevieve Smith, Caroline L Donovan

**Affiliations:** 1 Centre for Health Research & School of Psychology and Wellbeing University of Southern Queensland Education City Springfield Central Australia; 2 Manna Institute Springfield Australia; 3 Australian Institute of Suicide Research and Prevention Griffith University Brisbane Australia; 4 School of Applied Psychology & Centre for Mental Health Griffith University Mt Gravatt Australia; 5 Centre for Health Research University of Southern Queensland Springfield Australia

**Keywords:** internet-delivered cognitive behavioral therapy, ICBT, anxiety, child, adolescent, stepped care, videoconferencing

## Abstract

**Background:**

Self-guided internet-delivered cognitive behavioral therapy (ICBT) achieves greater reach than ICBT delivered with therapist guidance, but demonstrates poorer engagement and fewer clinical benefits. Alternative models of care are required that promote engagement and are effective, accessible, and scalable.

**Objective:**

This randomized trial evaluated whether a stepped care approach to ICBT using therapist guidance via videoconferencing for the step-up component (ICBT-SC[VC]) is noninferior to ICBT with full therapist delivery by videoconferencing (ICBT-TG[VC]) for child and adolescent anxiety.

**Methods:**

Participants included 137 Australian children and adolescents aged 7 to 17 years (male: n=61, 44.5%) with a primary anxiety disorder who were recruited from participants presenting to the BRAVE Online website. This noninferiority randomized trial compared ICBT-SC[VC] to an ICBT-TG[VC] program, with assessments conducted at baseline, 12 weeks, and 9 months after treatment commencement. All ICBT-TG[VC] participants received therapist guidance (videoconferencing) after each session for all 10 sessions. All ICBT-SC[VC] participants completed the first 5 sessions online without therapist guidance. If they demonstrated response to treatment after 5 sessions (defined as reductions in anxiety symptoms to the nonclinical range), they continued sessions without therapist guidance. If they did not respond, participants were stepped up to receive supplemental therapist guidance (videoconferencing) for the remaining sessions. The measures included a clinical diagnostic interview (Anxiety Disorders Interview Schedule) with clinician-rated severity rating as the primary outcome and parent- and child-reported web-based surveys assessing anxiety and anxiety-related interference (secondary outcomes).

**Results:**

Although there were no substantial differences between the treatment conditions on primary and most secondary outcome measures, the noninferiority of ICBT-SC[VC] compared to ICBT-TG[VC] could not be determined. Significant clinical benefits were evident for participants in both treatments, although this was significantly higher for the ICBT-TG[VC] participants. Of the 89 participants (38 in ICBT-SC[VC] and 51 in ICBT-TG[VC]) who remained in the study, 26 (68%) in ICBT-SC[VC] and 45 (88%) in ICBT-TG[VC] were free of their primary anxiety diagnosis by the 9-month follow-up. For the intention-to-treat sample (N=137), 41% (27/66) ICBT-SC[VC], and 69% (49/71) ICBT-TG[VC] participants were free of their primary anxiety diagnosis. Therapy compliance was lower for the ICBT-SC[VC] participants (mean 7.39, SD 3.44 sessions) than for the ICBT-TG[VC] participants (mean 8.73, SD 3.08 sessions), although treatment satisfaction was moderate to high in both conditions.

**Conclusions:**

This study provided further support for the benefits of low-intensity ICBT for children and adolescents with a primary anxiety disorder and highlighted the excellent treatment outcomes that can be achieved through therapist-guided ICBT delivered via videoconferencing. Although noninferiority of the stepped care adaptive approach could not be determined, it was acceptable to families, produced good outcomes, and could assist in increasing access to evidence-based care.

**Trial Registration:**

Australian New Zealand Clinical Trials Registry (ANZCTR) ACTRN12618001418268; https://anzctr.org.au/Trial/Registration/TrialReview.aspx?ACTRN=12618001418268

## Introduction

### Background

Anxiety disorders affect approximately 15% to 20% of children and adolescents, with symptoms and impacts (eg, impairment in academic, social, and family functioning) persisting without treatment [1]. Therefore, it is important to identify effective treatments that can be made widely accessible. Cognitive behavioral therapy (CBT) delivered through a face-to-face (F2F) modality has been well validated as an effective treatment for anxiety in young people [2] but involves substantial and costly therapist contact, with many young people being unable to access such treatments [3]. Internet-delivered CBT (ICBT) has been identified as a viable alternative for youth anxiety in several randomized controlled trials (RCTs) [2,4-6]. However, it is noted in meta-analytic reviews that the effects of ICBT are dependent on the level of therapist support or guidance provided during treatment [2,7]. Therapist-guided ICBT (ICBT-TG), where regular but minimal therapist guidance is provided in addition to an ICBT program, has demonstrated similar effects to F2F CBT and seems to promote greater treatment engagement and response than self-directed ICBT interventions [2,8,9]. While ICBT-TG may enhance engagement and produces good therapeutic outcomes, its widespread delivery requires the funding of therapist time and is constrained by issues of workforce shortages. By contrast, self-directed ICBT has the potential to reach thousands of young people and has been shown to produce moderate improvements in youth anxiety; yet, engagement with self-directed interventions is less than ideal, and, consequently, treatment effects are not optimal for all [9,10]. Alternative ICBT models are required that promote engagement and are effective, accessible, and scalable.

### Stepped Care and Adaptive Interventions

Stepped care and adaptive interventions are potential alternative treatment models, where intervention type, modality, and intensity are adjusted based on treatment need or individual characteristics at various points during or after treatment steps [11]. Stepped care approaches aim to achieve a balance between intervention intensity and clinical effectiveness by “stepping up” intervention intensity only for those young people who need it. In stepped care models, all participants receive the same least intensive intervention first. Progress is then evaluated, and only those who need additional support are transitioned to a more intensive intervention [12,13]. Traditionally, in child anxiety, the evaluation of progress has occurred at the end of a treatment block, such as after the completion of an ICBT program (8-12 sessions; step 1), with children stepped up to further treatment (eg, F2F CBT) in the second step if required [14,15]. Such stepped care models have shown similar efficacy to a standard full program of F2F CBT [14]; yet, they offer greater efficiency in that not all young people need to progress to the more intensive and costly second step. However, stepped care delivered in this way means that those who are “stepped up” are required to progress through multiple treatment steps (or full programs) before they experience treatment success, risking disengagement with treatment and the unnecessary duplication of treatment content [11,16].

Adaptive interventions are a recent advancement within ICBT that involve adjusting treatment content or modality during the intervention (rather than at the end) in response to progress monitoring or identification of “risk” or “treatment need” [16]. Such adaptive interventions, which have thus far primarily been examined in adult populations, bring forward the “step-up” point to identify problems early and optimize the care received. The work of Forsell et al [16] has demonstrated that algorithms implemented at week 4 of treatment to predict individual outcomes can successfully redirect patients at risk into an adapted treatment and produce improved treatment outcomes compared to those who received a standard treatment. Treatment adaptations can include changes to the level of support, types of materials and messaging content provided (as examined by Forsell et al [16]), or actual treatment content as required and at multiple time points. Although still in an early stage, adaptive interventions may offer promise in ICBT for child and adolescent anxiety, where problems of poor early engagement and subsequent reduced outcomes have been identified in low-intensity, self-directed ICBT [9,10].

Previously, our team trialed a “stepped care adaptive” approach to ICBT for young people with anxiety [11], where children and adolescents with an anxiety disorder were randomized to either “stepped care ICBT” (ICBT-SC) or ICBT-TG. Those allocated to the latter condition received ICBT-TG in the form of minimal weekly email support from a therapist from the beginning of treatment. All young people in the ICBT-SC condition commenced the low-intensity, self-guided ICBT as the “first step” or treatment block. After 5 sessions (treatment midpoint), a self-report assessment and associated clinical judgment process was integrated (rather than a computer algorithm) to determine participant progress and need for stepping up (adapting treatment). Those who had not demonstrated adequate treatment gains after 5 sessions (low engagement and limited response) were stepped up to receive therapist guidance (in the form of weekly email support) for the remainder of treatment. Those who demonstrated good engagement and treatment response (based on clinical cutoffs in self-report measures) continued with the lower-intensity, self-directed ICBT intervention. ICBT-SC was found to be acceptable to families and noninferior to ICBT-TG at the 12-week and 9-month follow-ups, with significant clinical benefits evident for participants in both treatment conditions. At the 9-month follow-up, 77% of the young people in both conditions were free of their primary diagnosis. In this previous trial, the therapist guidance provided was low in intensity (email support), preserving the potential scalability and accessibility of the intervention.

Thus, low-intensity, stepped care ICBT approaches that integrate their “step-up” or “adaptive” point during treatment may represent a suitable way of providing ICBT that is more easily accessible to young people. However, additional research is required, especially with young people, to establish a clear evidence base and determine ideal treatment adaptations and modalities. While therapist guidance delivered by messaging, as in the study by March et al [11], can be effective, many young people and parents continue to demonstrate a preference for at least some occasional F2F meetings within ICBT [17,18]. Furthermore, in a recent systematic review, Vu and Per [18] demonstrated an average treatment session completion rate of only 72% for asynchronous technology–based CBT (eg, email supported), highlighting potential problems with engagement. Thus, there is a clear need to provide alternative treatment models that address such factors. Recent shifts in health care delivery highlight new modalities that could be integrated into stepped care and adaptive treatment models. Videoconferencing, in particular, has emerged as an important technology that can be used as a form of F2F delivery of CBT programs [19,20] or as a way of providing minimal therapist guidance to support ICBT [21].

### Videoconferencing

Videoconferencing is increasingly being implemented to overcome treatment gaps, especially since the COVID-19 pandemic [22], and refers to the integrated use of audio and video content for live communication and treatment delivery. Videoconferencing differs from other forms of therapist communication, such as the email therapist guidance provided in the study by March et al [11], which operates as an asynchronous, delayed communication method. It allows opportunities for the young person and therapist to meet remotely in real time and communicate synchronously. Typically, videoconferencing requires participants to set a regular time to meet their therapist, allows the opportunity for rapport to be developed, and enables the therapist to discuss treatment techniques with the young person directly as well as observe nonverbal and other reactions that could indicate potential challenges to the implementation of the strategies taught. It also allows the therapist to use the technological functions of videoconferencing software, such as screen sharing, as a method of hands-on demonstration, clarification of material, and consolidation of learning [21]. Furthermore, videoconferencing may appeal to young people with anxiety for whom attending in-person sessions, traveling, and being in public areas can be challenging and anxiety provoking [20]. Despite the common use of videoconferencing in the delivery of psychological therapy, there is very little investigation of its efficacy either as an adjunct for ICBT or on its own as a delivery mode for CBT, particularly for child and adolescent anxiety.

A recent systematic review examined the outcomes for F2F CBT delivered via videoconferencing for child and adolescent anxiety [22]. The review found that in the scarce literature available (a pooled sample of only 213 young people), significant reductions in anxiety outcomes were evident for videoconferencing CBT after treatment, with moderate to large effect sizes. Importantly, average treatment completion was reported as 89%, higher than that reported in the review by Vu and Per [18] of asynchronous technology–based CBT and highlighting the potential of videoconferencing in promoting treatment engagement. However, it is important to note that most of the studies in this review had small sample sizes and failed to include a control group or report on satisfaction, treatment completion, or treatment fidelity [22]. Furthermore, the review was unable to make distinctions regarding the value of videoconferencing when used as an adjunct to ICBT or as the mode of full delivery of CBT. Thus, videoconferencing seems to offer promise in providing therapist guidance for ICBT interventions for young people with anxiety and could hold particular value for stepped care or adaptive interventions. This study was based on the premises that therapist guidance (the support) itself is the key element of evidence-based ICBT, and that when delivered via videoconferencing (essentially F2F support), this would also be effective.

### The Aims of This Study

This study represents the first RCT examining the impact of an ICBT-SC model for children and adolescents with a primary anxiety disorder in which the therapist-guided element is provided using videoconferencing (VC). Specifically, this noninferiority RCT examines whether a stepped care version of ICBT using therapist guidance via videoconferencing for the step-up component (ICBT-SC[VC]) can produce the same benefits for young people as evidence-based ICBT with therapist guidance provided throughout the whole treatment via videoconferencing (ICBT-TG[VC]). It was hypothesized that ICBT-SC[VC] would be no less clinically effective than ICBT-TG[VC], that is, not inferior to any greater extent than the noninferiority margin, with similar reductions in anxiety severity from baseline to 12 weeks and 9 months after baseline assessment. It was also predicted that the ICBT-SC[VC] program would be acceptable and satisfactory to participants.

## Methods

### Design

The design involved a 2-arm noninferiority RCT following CONSORT (Consolidated Standards of Reporting Trials) guidelines [23] comparing ICBT-SC[VC] with evidence-based ICBT-TG[VC]; detailed information is presented in the Interventions subsection [5,24,25]. The results are presented according to the CONSORT-EHEALTH (Consolidated Standards of Reporting Trials of Electronic and Mobile Health Applications and Online Telehealth) checklist (Multimedia Appendix 1).

Stratified block random assignment was used to control for participant age (7-12 y and 13-17 y), with an allocation ratio of 1:1 to treatment conditions. A randomization table was created using computer-generated sequences. The lead investigator generated the random sequence, which was concealed from the project manager using opaque envelopes until each individual participant was ready to be randomized. Full diagnostic and web-based questionnaire assessments were conducted before the intervention (baseline), at 12 weeks, and 9 months (primary end point) after the commencement of treatment. At midtreatment (after session 5 or week 7, whichever occurred first), a brief assessment was conducted to determine “responder status” and the “step-up” decision.

### Ethical Considerations

The study was conducted in accordance with the University of Southern Queensland Human Ethics Research Committee (H17REA271). The study design, hypotheses, and analysis plan were preregistered with the Australian New Zealand Clinical Trials Registry (ACTRN12618001418268) and reported in line with international recommendations for child anxiety trials [26]. All participants who met the inclusion criteria provided informed consent (parent and child).

### Participants and Procedure

Participants included 137 young people (male: n=61, 44.5%; female: n=76, 55.5%) aged 7 to 17 (mean 10.93, SD 2.26) years, who registered for the BRAVE Online program (an ICBT self-guided intervention for child and adolescent anxiety) between August 30, 2018, and August 26, 2019, and met the inclusion criteria outlined in the next paragraph. Participants were directed to the program through self-referral or referral by general practitioners, education professionals, or mental health professionals.

Participants were required to meet the following criteria to be included in the study: be aged between 7 and 17 years; have elevated levels of anxiety on the Children’s Anxiety Scale-8 (CAS-8) [27]; have a primary diagnosis of social anxiety disorder, generalized anxiety disorder, separation anxiety, or specific phobia on the Anxiety Disorders Interview Schedule for Children–Child version (ADIS-C) [28]; have access to the BRAVE Online program via a computer or mobile device with an Australian IP address; and be able to read and write English at an age-appropriate level. Participants with a primary diagnosis of obsessive-compulsive disorder, posttraumatic disorder, and panic disorder were not eligible for this study because these are not addressed in the treatment program, although these diagnoses were permitted if secondary to the primary presenting issue. Participants with secondary mood disorders were included, providing that their mood disturbance was rated <6 on the ADIS-C (refer to the Primary Outcome Measures subsection). Participants were excluded from the study if they exhibited current suicidal ideation, self-harm behaviors, substance abuse problems, significant behavioral disorders, pervasive developmental disorders, or learning disorders as assessed during the clinical interview. In addition, those already receiving support from a professional were excluded.

The 396 participants who initially enrolled in the BRAVE Online self-help program and reported elevated anxiety on a routine registration questionnaire (refer to Participant Selection Measures section), were invited to participate in an initial telephone screening assessment. Of the 396 participants, 117 (29.5%) declined the invitation or were unable to be contacted, 106 (26.8%) did not meet the broad inclusion criteria at this stage (eg, already receiving support from a professional), and 173 (43.7%) were invited to participate in a more detailed assessment to determine inclusion criteria, involving baseline web-based questionnaires and a telephone diagnostic interview (with the young person; refer to the Primary Outcome Measures subsection). After this assessment, of the 173 participants, 27 (15.6%) were excluded because they did not demonstrate a clinical-level anxiety disorder or had a primary disorder other than anxiety, and 9 (5.2%) declined further participation. Thus, of the initially enrolled 396 participants, 137 (34.6%) met the inclusion criteria, provided informed consent (parent and child), and were randomly allocated to a treatment condition. Of these 137 participants, 97 (70.8%) had a comorbid anxiety disorder at a clinical level, with an average of 2.05 (SD 0.85) anxiety diagnoses. Only 2 (1.5%) of the 137 participants had a comorbid mood disorder (dysthymia).

### Measures

#### Demographics

Basic demographic information, including age, gender, and residential location, was collected during program registration. Postcode data were coded according to the Australian Statistical Geography Standard [29], and participants were categorized as residing in major cities or inner regional, outer regional, or remote locations. Postcode data were also used to ascertain area-level socioeconomic status through the Socio-Economic Indexes for Areas [30], reported by quartile.

#### Participant Selection Measures

In addition to the ADIS-C diagnostic interview described in the next subsection, the CAS-8 [27] was completed by young people when registering for the BRAVE Online program to enable us to identify those with elevated levels of anxiety for inclusion in the study. The CAS-8 is a brief 8-item measure based on the Spence Children’s Anxiety Scale (refer to Secondary Outcome Measures section). It has demonstrated excellent reliability, including in online delivery formats [26]. Population-level, gender-standardized norms are provided, with scores above the 84th percentile considered elevated (T-scores >60: CAS-8 scores ≥12 for female individuals and ≥10 for male individuals).

#### Primary Outcome Measures

The presence and type of an anxiety disorder were determined using the ADIS-C [28] and the associated clinician severity rating (CSR; primary outcome measure) ranging from 0 (none) to 8 (severely disturbing or disabling), which was the primary outcome measure in this study. The ADIS-C was administered to the child via telephone with young people only at baseline, 12 weeks, and 9 months by trained interviewers who were blinded to the experimental condition, study procedures and hypotheses and were supervised by an experienced psychologist. Only the child version of the ADIS was administered in this study to minimize participant burden and obtain information directly from the person completing treatment (no parent involvement in treatment). For participants aged <12 years, parents were given the choice to either attend the interview with their child or be provided with a summary upon completion of the telephone call.

Data from the ADIS-C also informed the clinician rating of overall child functioning on the Children’s Global Assessment Scale (CGAS) [31]. Scores ranged from 1 to 100, with the 81-to-100 band representing normal levels of functioning, 61 to 80 indicating slight disability, 41 to 60 representing moderate disability, and 1 to 40 indicating serious disability. The CGAS was a secondary outcome measure and has good interrater and test-retest reliability [31,32]. A sample of interviews (15%) were randomly selected to be recorded and coded for interrater reliability by a second assessor from the pool of assessors. Analysis revealed an intraclass coefficient of 0.99 for diagnosis type (*r*=0.96 for CSR ratings and *r*=0.94 for CGAS ratings).

#### Secondary Outcome Measures

Secondary outcome measures were assessed via web-based survey within 2 weeks of registration. The full Spence Children’s Anxiety Scale–Child and Parent versions (SCAS-C and SCAS-P, respectively) [33,34] were administered at baseline, midtreatment, 12 weeks, and 9 months as a secondary outcome measure and to determine “step-up” decisions at midintervention (refer to Determining Responder Status at Midtreatment for the Stepped Care Condition section). The internal consistency values in this study were Cronbach α=0.86 (SCAS-C) and Cronbach α=0.88 (SCAS-P).

The Child Anxiety Life Interference Scale–Child and Parent reports (CALIS-C and CALIS-P, respectively) [35] were used to assess the level of anxiety-induced life interference and impairment experienced by the child (as reported by children and parents) and by the parents themselves (parent report) at baseline, 12 weeks, and 9 months. The average internal consistency values for the CALIS-C were Cronbach α=0.84 (outside the home) and Cronbach α=0.70 (at home) and for the CALIS-P were Cronbach α=0.83 (outside the home), Cronbach α=0.76 (at home) and Cronbach α=0.88 (parent life).

#### Program Adherence and Satisfaction

Program adherence was measured by the number and proportion of sessions completed at 12 weeks and 9 months. Dropout status was determined by whether the participant withdrew from the study (treatment and assessments), recorded as *yes* or *no* at each assessment time point (midpoint, 12 wk, and 9 mo). Program satisfaction was measured after 3, 6, and 9 sessions through a 5-item scale used in our prior research [9,24]. Participants were required to respond to items assessing whether they would tell a friend about the program (item 1), how helpful the program was (item 2), how happy they were with the program (item 3), how much the program helped to reduce their anxiety (item 4), and their overall judgment of the program (item 5). Responses were provided on a 5-point Likert scale, with item 1 scores ranging from 1=*definitely not* to 5=*definitely yes*; items 2, 3, and 4 scores ranging from 1=*not at all*, to 5=*very much*; and item 5 scores ranging from 1=*very bad* to 5=*very good*. A total satisfaction score was calculated by summing responses to the 5 questions. Total scores could range from 5 to 25 [9].

### Interventions

#### The ICBT-TG[VC] Intervention

The ICBT-TG[VC] intervention was the 10-session child or adolescent BRAVE Online program delivered online, supported by videoconferencing therapist guidance for all sessions. The BRAVE Online programs comprise ten 45-minute sessions of ICBT and have demonstrated efficacy across several trials with minimal therapist guidance provided by email or messaging [5,24,25]. The only difference for this study was that the minimal therapist guidance was delivered via videoconferencing rather than email or messaging. The BRAVE Online program includes self-guided sessions that the young person can complete on an internet-enabled device or computer at any time and from any place. Sessions are designed to be participative and include text, videos, stories, examples, quizzes, question-and-answer activities, and additional interactive worksheets. The first 5 sessions of the BRAVE Online program include evidence-based anxiety management strategies such as the recognition of symptoms, relaxation, coping self-talk, cognitive restructuring, and graded exposure, with the second half of the treatment (sessions 6-10) incorporating additional strategies such as problem-solving, self-reinforcement, relapse prevention, and ongoing skills rehearsal [36,37]. Young people complete the interactive sessions on a computer or a tablet device. There is also a parent program, but this was not used in this study. Participants receive automated reminders each week when their session is due.

Each participant was assigned a therapist who monitored their progress, viewed their responses to activities, and provided guidance after each session via videoconference (15 min of therapist time per session) at a predetermined time. Videoconferencing sessions provided support to the young person with content of the session and skill rehearsal. Videoconferencing sessions were delivered to the young person, but the parent had the option of being present. BRAVE Online program therapists used these 15-minute sessions to provide reinforcement of effort, redirection, and clarification of participant responses where required, as well as to use screen sharing when beneficial to expand on ICBT content. Participants also received one 30-minute video call after session 5 (instead of the 15-minute session) to help with the construction and implementation planning of their exposure hierarchy. Participants could contact the BRAVE Online program for technical or administrative support (although they were not encouraged to contact the clinician between sessions). Participants had access to a total of 165 minutes of therapist guidance across the 10-session program.

#### The ICBT-SC[VC] Intervention

The ICBT-SC[VC] condition was BRAVE Online stepped care, comprising 2 steps depending on the results of a midpoint assessment. In step 1, all participants commenced the first 5 sessions of the BRAVE Online program, which involved low-intensity, self-guided ICBT without therapist guidance. Participants were then assessed at the midtreatment point (after 5 sessions or 7 weeks, whichever occurred first) to determine their “responder status” as described in the next subsection. In step 2, participants who “responded” to step 1 completed the remaining 5 sessions of the BRAVE Online program on a self-guided basis without therapist guidance, whereas those who did not respond to step 1 (did not show sufficient improvements) were “stepped up” to complete the remaining sessions of the BRAVE Online program supplemented with therapist guidance. Therapist guidance for participants who were “stepped up” was identical to that provided for ICBT-TG[VC]). They received a 30-minute videoconferencing session after session 5 to assist with the implementation of exposure. Those who were not stepped up did not receive any videoconferencing therapist guidance. The ICBT program (BRAVE Online) content and therapeutic strategies were identical across conditions, with only the amount of therapist guidance differing. A more detailed description of the BRAVE Online stepped care program and treatment content can be found in the study by March et al [21].

#### Determining Responder Status at Midtreatment for the Stepped Care Condition

The process for determining responder status in this study mirrored the process used by the team in the previous trial of the BRAVE Online stepped care program [13]. This process and step-up rules were determined after pilot work by the team [21]. Two factors were considered by a clinical psychologist when determining a participant’s responder status at the midtreatment assessment point. First, responder status for the midtreatment “step-up” decision was primarily determined by response on the SCAS-C and SCAS-P [33,38], using gender-standardized cutoffs based on normative and clinical child and adolescent samples [38,39]. Scores above the 84th percentile (T-scores >60) were considered elevated. “Responders” were defined as those who demonstrated a reduction in anxiety to the nonelevated range on *either* their primary anxiety subscale *or* total anxiety scores on the SCAS-C or SCAS-P at midtreatment for the scales on which they were elevated at baseline. This criterion was selected to ensure that we captured participants who made any clinically meaningful change in terms of anxiety symptomatology because not all participants necessarily targeted their primary anxiety in the first 5 sessions. “Nonresponders” were defined as those who did not meet this criterion.

As demonstrated in pilot work [21] and a previous trial [11], the level of session adherence was not necessarily consistent with reductions in anxiety symptoms as reported via the SCAS-C and SCAS-P, and additional factors required consideration in the step-up decision. Thus, in line with the study by March et al [11], as a second step, the clinical psychologist scoring the midtreatment assessment also examined treatment adherence (the number of sessions completed by midtreatment) and the way in which the young person engaged with program activities (in-session responses). Completion of <3 sessions was deemed indicative of nonresponse, along with answers to program activities that showed little thought, insight, or strategy implementation. Completion of between 3 and 5 sessions, along with good engagement with sessions (regular session completion as well as evidence of skill rehearsal and practice) was considered indicative of good response, if accompanied by improvements on the SCAS-C or SCAS-P. Each step-up decision was reviewed by the senior psychologist and lead investigator before finalization. Following the protocol of March et al [11], midtreatment assessments (web-based survey results and administrative program data and program responses) were evaluated by a clinical psychologist and recommendations discussed with the lead investigator (SM) for each case to ensure that guidelines were adhered to.

Participants who did not respond to the invitation to complete the midtreatment assessment were reminded 1 week later, and the clinical psychologist attempted to make contact via telephone. Those participants who did not respond were able to continue with their existing program if they chose to. Of the 53 participants, 1 (1.9%) did not complete the midtreatment assessment but continued in the study.

#### Therapist Training

Therapists had completed a minimum of a 4-year undergraduate degree in psychology and were undergoing or had completed further psychology education at the master’s or doctoral level (postgraduate clinical training). All therapists received 4 hours of training in the BRAVE Online program and were provided with agenda templates for videoconference sessions. The therapists also participated in ongoing fortnightly supervision with a senior clinical psychologist.

### Analytic Strategy

The analytic strategy followed that of a previous noninferiority trial of the BRAVE Online program [13]. Noninferiority analyses were conducted for the clinician-rated and self-report measures. Following the procedure developed by Feingold [40], hierarchical linear models (HLMs) were used to estimate the changes over time and were converted to standardized measures (Cohen *d*)*.* The effects estimated included changes in primary and secondary outcomes for participants in the ICBT-SC[VC] and ICBT-TG[VC] conditions across time (from baseline to 12 weeks and from baseline to 9 months), as well as the interaction between treatment condition and time (if the rate of change was similar or different between conditions). HLM analysis was conducted using maximum likelihood estimation with the *nlme* package [36] in R [37]. CIs for effect sizes were determined as described by Zaiontz [41], using a noncentral T-distribution [42]. Noninferiority was supported when the lower limit of the 95% CI for the standardized mean difference was within the margin of noninferiority.

The margin of noninferiority (Δ) for the treatment effect was determined for the primary outcome measure (CSR derived from the ADIS-C) based on the results of previous trials comparing ICBT-TG to a waitlist control demonstrating effect sizes of Cohen *d*=0.60, Cohen *d*=1.22, and Cohen *d*=1.45 at the 12- to 14-week assessment [5,21,25]. It is recommended that a “clinically unimportant difference” between 2 treatments should be *one-half or less* of the effect size of the reference intervention [43]. Thus, the selection of the noninferiority margin was based on the previous reference effect of Cohen *d*=0.60, and for the primary CSR, the margin of noninferiority was set to Cohen *d*=0.20 such that if the lower bound of the 95% CI of the effect size did not exceed Cohen *d*=–0.40, ICBT-SC[VC] would be deemed as noninferior to ICBT-TG[VC]. This method was selected to follow that of another noninferiority trial conducted on the BRAVE Online program intervention [11]. The same margin of noninferiority was used across all secondary outcome measures. The power calculation was set at α=.05, for a lower-bound noninferiority margin of 0.4, to provide a power of 0.80 for the primary and secondary outcomes, requiring a sample size of 58 participants per condition. The aim was to recruit 66 participants per group, given an expected attrition rate of 15%. The HLM approach allows for all data to be included in the analysis, including data of participants who drop out, aligning with an intention-to-treat (ITT) approach.

To examine the clinical benefits obtained by participants, diagnostic outcomes were compared between conditions using chi-square analyses and logistic regression analyses for both completer and ITT samples. Odds ratios (ORs; ICBT-SC[VC] as the baseline condition) and 95% CIs were calculated. The ITT sample for the chi-square analyses was determined using the last-observation-carried-forward method for participants who withdrew from the study or failed to complete assessment points for diagnostic outcomes only.

## Results

### Participant Characteristics

A summary of baseline participant characteristics, split by treatment condition, is presented in Table 1.

**Table 1:** Baseline sample characteristics (n=137).

Characteristics		ICBT-SC[VC]^a^, n=66	ICBT-TG[VC]^b^, n=71
**Gender, n (%)**			
	Male	28 (42)	33 (46)
	Female	38 (58)	38 (54)
**Geographic location, n (%)**			
	In major city	46 (70)	38 (54)
	Out of major city	11 (17)	23 (32)
	Missing	9 (14)	10 (14)
**SEIFA^c,d^ (percentile), n (%)**			
	0-25th	6 (9)	9 (13)
	26th-50th	11 (17)	14 (20)
	51st-75th	19 (29)	16 (23)
	76th-100th	27 (41)	30 (42)
	Missing	3 (5)	2 (3)
**Primary diagnosis, n (%)**			
	Generalized anxiety disorder	37 (56)	42 (59)
	Separation anxiety disorder	9 (14)	11 (15)
	Social phobia	12 (18)	8 (11)
	Specific phobia	8 (12)	10 (14)
Age (y), mean (SD)		11.08 (2.35)	10.79 (2.18)
Treatment expectancies, mean (SD)		25.84 (8.06)	28.22 (7.56)
CSR^e^ for primary anxiety diagnosis, mean (SD)		5.42 (0.91)	5.42 (0.87)
CGAS^f^, mean (SD)		54.77 (5.16)	54.31 (4.99)
Diagnoses (n), mean (SD)		1.94 (0.80)	2.15 (0.89)

^a^ICBT-SC[VC]: stepped care internet-delivered cognitive behavioral therapy with step-up component delivered with therapist guidance via videoconferencing.

^b^ICBT-TG[VC]: internet-delivered cognitive behavioral therapy with full therapist delivery by videoconferencing.

^c^SEIFA: Socio-Economic Index for Areas.

^d^Percentages may not add up to 100 due to rounding.

^e^CSR: clinician severity rating.

^f^CGAS: Children’s Global Assessment Scale.

### Program Adherence and Dropout

**Figure 1:**
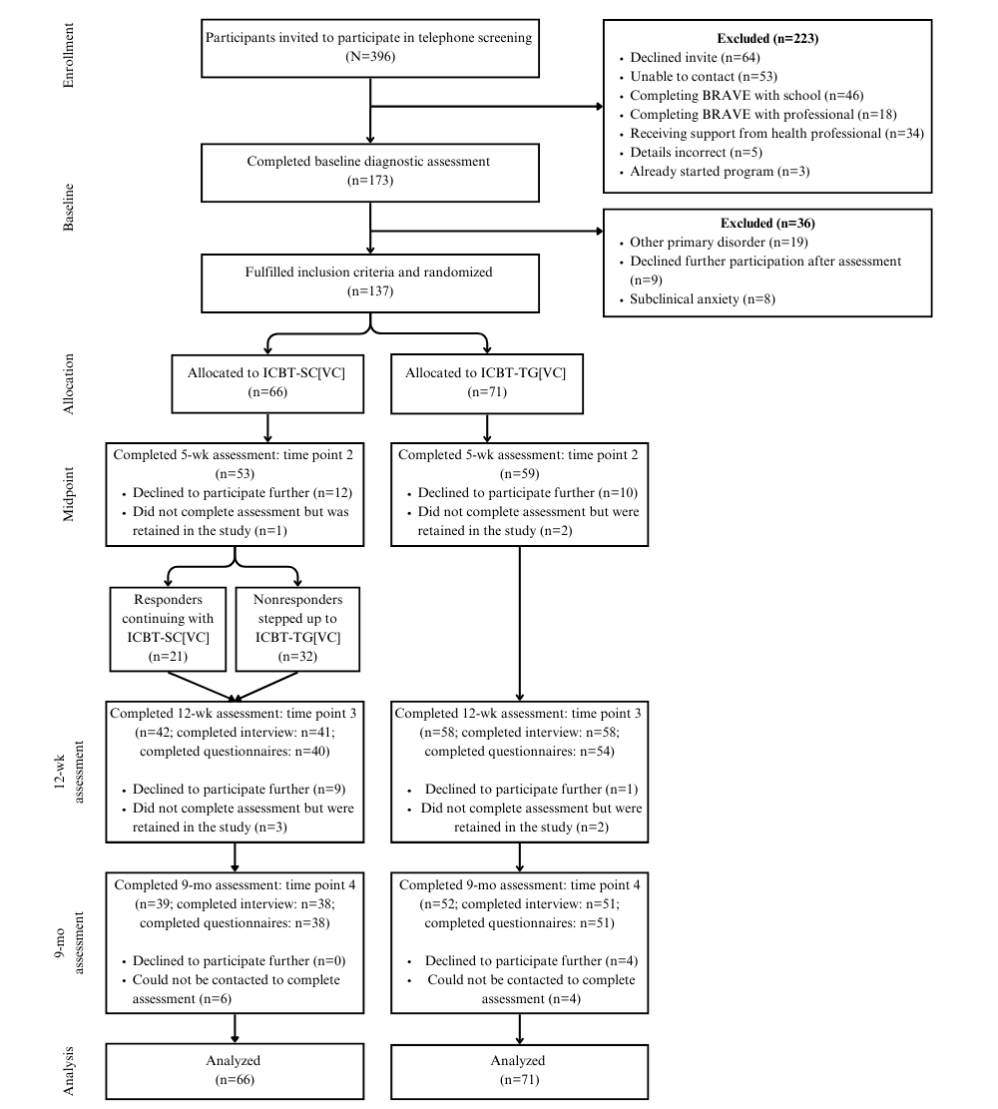
Flow of participants through the study. “Completed assessment” includes participants who completed any of the assessment items at that time point. Some of the participants completed interviews but not questionnaires, while some of the participants completed questionnaires but not interviews. ICBT-SC[VC]: stepped care internet-delivered cognitive behavioral therapy with step-up component delivered with therapist guidance via videoconferencing; ICBT-TG[VC]: internet-delivered cognitive behavioral therapy with full therapist guidance delivered by videoconferencing.

The flow of participants through each phase of the study is presented in Figure 1. At midpoint, significantly more sessions were completed by participants in the ICBT-TG[VC] condition (mean 4.48, SD 1.50) than those in the ICBT-SC[VC] condition (mean 3.18, SD 1.62; t_135_=–4.87; *P*<.001; Cohen *d*=–0.83, 95% CI –1.18 to –0.48). By the 12-week assessment, ICBT-TG[VC] participants had completed more sessions (mean=7.72, SD 3.06) than the ICBT-SC[VC] participants (mean 5.61, SD 3.03; t_135_=–4.05; *P*<.001; Cohen *d*=–0.69, 95% CI –1.04 to –0.35). Similarly, at 9 months, ICBT-TG[VC] participants had completed more sessions (mean 8.73, SD 3.08) than ICBT-SC[VC] participants (mean 7.39, SD 3.44; t_135_=–2.40; *P*<.001; Cohen *d*=–0.41, 95% CI –0.75 to –0.07). Multimedia Appendix 2 reports the proportion of participants completing each of the 10 sessions of the program by condition. No adverse effects were reported to the investigators or ethics committee.

The proportion of participants who dropped out of the study (refused to engage in any further treatment or assessments) was not significantly different by condition at the midtreatment point (OR 1.29, 95% CI 0.59-2.79; *P*=.51; 12/66, 18% vs 10/71, 14% for ICBT-SC[VC] vs ICBT-TG[VC]), respectively) or by 9 months (OR 1.51, 95% CI 0.85-2.67; *P*=.16; 21/66, 32% vs 15/71, 21% for ICBT-SC[VC] vs ICBT-TG[VC], respectively). However, significantly more participants had dropped out of ICBT-SC[VC] (21/66, 32%) than ICBT-TG[VC] (11/71, 16%) at the 12-week assessment (OR 2.05, 95% CI 1.07-3.93; *P*=.02).

### Proportion of Participants Stepped Up

Of the 53 ICBT-SC[VC] participants, 32 (60%) were classified as “nonresponders” at the midpoint assessment and were subsequently stepped up to ICBT-TG[VC], while 21 (40%) were classified as “responders” and continued with self-guided sessions.

### Noninferiority Analysis

**Figure 2:**
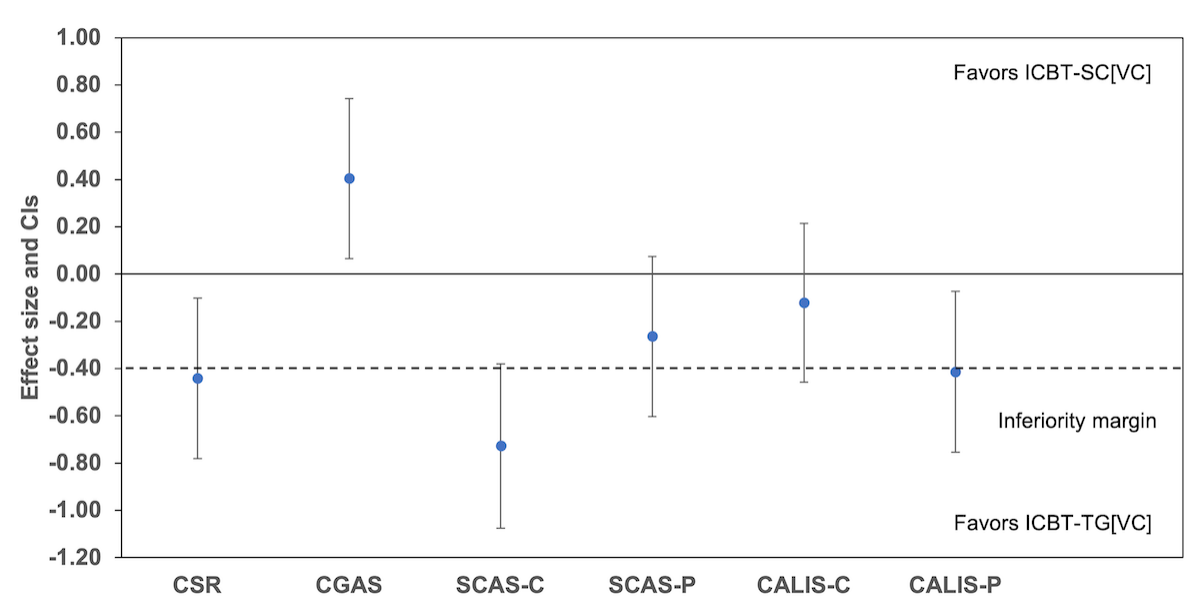
The 95% CIs for the effect size (Cohen d) for the difference between stepped care internet-delivered cognitive behavioral therapy with step-up component delivered with therapist guidance via videoconferencing (ICBT-SC[VC]) and ICBT with full therapist guidance delivered by videoconferencing (ICBT-TG[VC]) in changes in mean scores from baseline to 12 weeks for clinician-, child-, and parent-reported outcomes. CALIS-C: Child Anxiety Life Interference Scale–Child report; CALIS-P: Child Anxiety Life Interference Scale–Parent report; CGAS: Children’s Global Assessment Scale; CSR: Clinician Severity Rating; SCAS-C: Spence Children’s Anxiety Scale–Child version; SCAS-P: Spence Children’s Anxiety Scale–Parent version. Positive values favor ICBT-SC[VC] except for the CGAS for which negative effect size favors ICBT-SC[VC].

**Figure 3:**
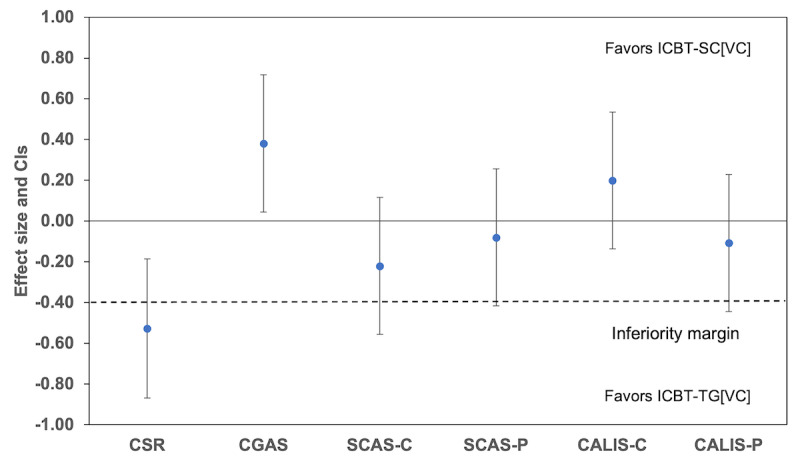
The 95% CIs for the effect size (Cohen d) for the difference between stepped care internet-delivered cognitive behavioral therapy with step-up component delivered with therapist guidance via videoconferencing (ICBT-SC[VC]) and ICBT with full therapist guidance delivered by videoconferencing (ICBT-TG[VC]) in changes in mean scores from baseline to 9 months for clinician-, child-, and parent-reported outcomes. CALIS-C: Child Anxiety Life Interference Scale–Child report; CALIS-P: Child Anxiety Life Interference Scale–Parent report; CGAS: Children’s Global Assessment Scale; CSR: Clinician Severity Rating; SCAS-C: Spence Children’s Anxiety Scale–Child version; SCAS-P: Spence Children’s Anxiety Scale–Parent version. Positive values favor ICBT-SC[VC] except for the CGAS for which negative effect size favors ICBT-SC[VC].

The difference in the rate of change between the ICBT-TG[VC] and ICBT-SC[VC] conditions from baseline to 12 weeks and from baseline to 9 months can be seen in Figures 2 and 3 and Multimedia Appendix 3. The test of noninferiority, examining the difference between the conditions in the rate of change over time, indicated that the standardized 95% CIs for Cohen *d* exceeded the range of noninferiority for all measures from baseline to 12 weeks (Figure 2), favoring ICBT-TG[VC]. From baseline to 9 months (Figure 3), the standardized 95% CIs for Cohen *d* exceeded the range of noninferiority for all measures except for the CALIS-C. The ICBT-SC[VC] condition showed a slightly greater reduction in the CALIS-C scores compared to the ICBT-TG[VC] condition on this measure, but the difference was not statistically significant.

### Clinician- and Self-Reported Outcomes

**Figure 4:**
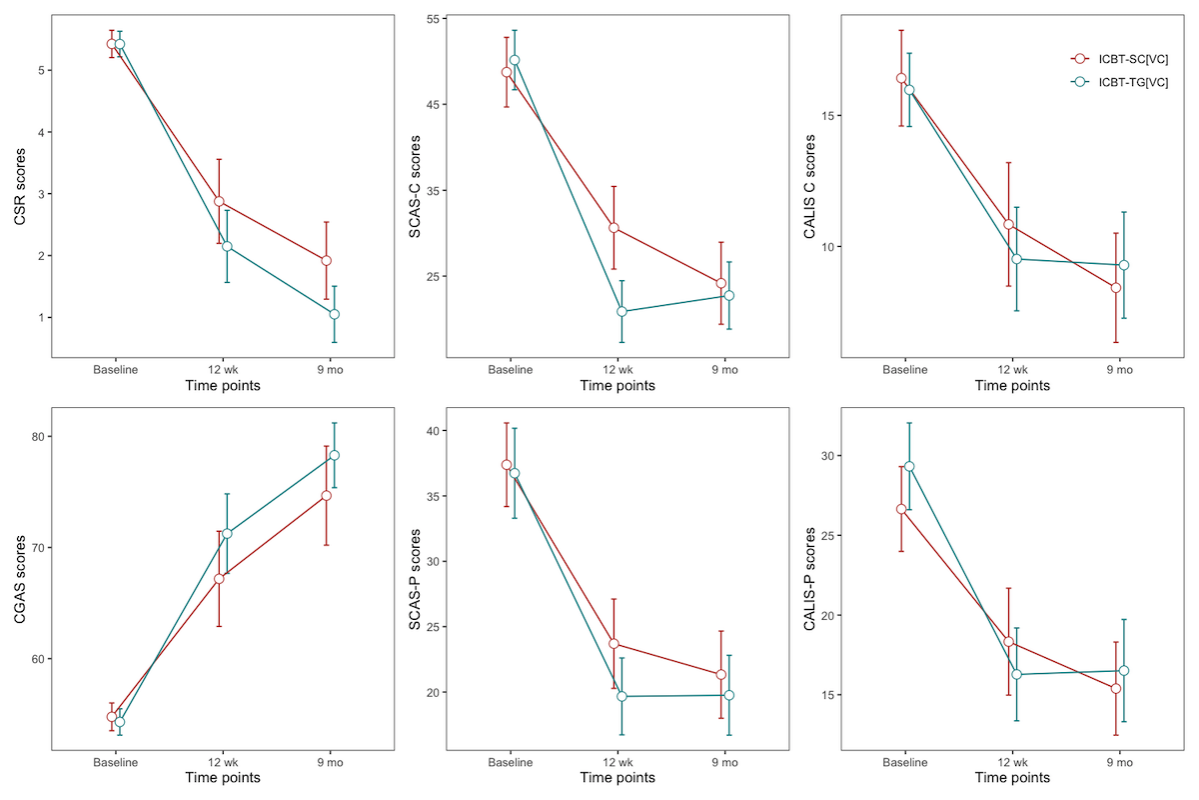
Changes in outcome measures across the time points. CALIS-C: Child Anxiety Life Interference Scale–Child report; CALIS-P: Child Anxiety Life Interference Scale–Parent report; CGAS: Children’s Global Assessment Scale; CSR: Clinician Severity Rating; ICBT-SC[VC]: stepped care internet-delivered cognitive behavioral therapy with step-up component delivered with therapist guidance via videoconferencing; ICBT-TG[VC]: internet-delivered cognitive behavioral therapy with full therapist guidance delivered by videoconferencing; SCAS-C: Spence Children’s Anxiety Scale–Child version; SCAS-P: Spence Children’s Anxiety Scale–Parent version.

To examine the changes in symptomatology by the ICBT-SC[VC] and ICBT-TG[VC] conditions, the results from the fixed effects from the HLM analyses are presented in Multimedia Appendix 4. Significant improvements across time were evident for all outcome measures for both intervention groups, and there were no significant differences between the conditions in general. The only significant time-by-condition interaction was for the SCAS-C, for which post hoc tests indicated a significantly greater reduction in scores from baseline to 12 weeks for the ICBT-TG[VC] condition compared to the ICBT-SC[VC] condition (t_113_=–3.16; *P*=.002). Table 2 reports the within-participant changes from baseline to 12 weeks and from 12 weeks to 9 months for each condition to be consistent with other studies of CBT for anxiety that report the treatment and follow-up periods separately. The trajectories of change are shown in Figure 4 (with error bars representing 95% CIs), and the estimated marginal means and SEs for child-, parent-, and clinician-reported outcomes at each assessment occasion for each condition are shown in Multimedia Appendix 4, along with between-condition effects at each time point. The rate of change from baseline to 9 months (Multimedia Appendix 3) was not significantly different between conditions on any measure other than the CSR, for which ICBT-TG[VC] showed a greater reduction than ICBT-SC[VC].

**Table 2:** Within-group treatment effects for each condition from baseline to 12 weeks and 12 weeks to 9 months.

Scale	ICBT-SC[VC]^a^					ICBT-TG[VC]^b^			
	Baseline to 12 wk, Cohen *d* (95% CI)^c^	*P* value	12 wk to 9 mo, Cohen *d* (95% CI)^c^	*P* value	Baseline to 12 wk, Cohen *d* (95% CI)^c^		*P* value	12 wk to 9 mo, Cohen *d* (95% CI)^c^	*P* value
CSR^d^	2.86 (2.11 to 3.61)	<.001	1.08 (0.25 to 1.90)	.01	3.68 (3.04 to 4.32)		<.001	1.23 (0.57 to 1.90)	<.001
CGAS^e^	–2.45 (–3.26 to –1.64)	<.001	–1.48 (–2.24 to –0.71)	<.001	–3.34 (–4.01 to –2.68)		<.001	–1.39 (–1.97 to –0.81)	<.001
SCAS-C^f^	1.15 (0.80 to 1.51)	<.001	0.41 (0.16 to 0.66)	.001	1.86 (1.58 to 2.15)		<.001	–0.12 (–0.31 to 0.07)	.23
SCAS-P^g^	1.00 (0.74 to 1.25)	<.001	0.17 (–0.06 to 0.41)	.15	1.24 (0.99 to 1.49)		<.001	–0.01 (–0.20 to 0.18]	.95
CALIS-C^h^	0.83 (0.40 to 1.26)	<.001	0.36 (0.02 to 0.70)	.04	0.96 (0.64 to 1.27)		<.001	0.03 (–0.29 to 0.36)	.83
CALIS-P^i^	0.74 (0.45 to 1.03)	<.001	0.26 (–0.02 to 0.54)	.07	1.16 (0.89 to 1.43)		<.001	–0.02 (–0.33 to 0.28)	.89

^a^ICBT-SC[VC]: stepped care internet-delivered cognitive behavioral therapy with step-up component delivered with therapist guidance via videoconferencing.

^b^ICBT-TG[VC]: internet-delivered cognitive behavioral therapy with full therapist delivery by videoconferencing.

^c^Positive Cohen *d* values indicate improvement over time, except for the Children’s Global Assessment Scale scores in which negative values indicate improvement over time (estimated from hierarchical linear modeling).

^d^CSR: clinician severity rating.

^e^CGAS: Children’s Global Assessment Scale.

^f^SCAS-C: Spence Children’s Anxiety Scale–Child version.

^g^SCAS-P: Spence Children’s Anxiety Scale–Parent version.

^h^CALIS-C: Child Anxiety Life Interference Scale–Child report.

^i^CALIS-P: Child Anxiety Life Interference Scale–Parent report.

### Diagnostic Outcomes

Diagnostic outcomes are presented in Table 3. The proportion of participants free of their primary and any anxiety diagnosis increased over time for both conditions. For the per-protocol analysis, there were no significant differences between the conditions in the proportion of participants free of their *primary* or *any* anxiety diagnosis at 12 weeks, although by 9 months, a significantly higher proportion of participants in the ICBT-TG[VC] condition were free of their primary and any anxiety diagnosis. For the ITT analysis, the proportion of participants free of their *primary* and *any* diagnosis was significantly higher for ICBT-TG[VC] than for ICBT-SC[VC] at both 12 weeks and 9 months. Of those ICBT-SC[VC] participants in the per-protocol sample who were stepped up to receive therapist guidance, 41% (9/22) were free of their primary diagnosis at 12 weeks compared to 68% (13/19) of those who were not stepped up (ie, continued to receive self-guided sessions). At 9 months, 55% (11/20) of those stepped up compared to 83% (15/18) of those not stepped up were free of their primary diagnosis.

**Table 3:** Diagnostic outcomes for each condition.

Time point and sample		Free of primary anxiety diagnosis					Free of any anxiety diagnosis				
		ICBT-SC[VC]^a^, n/N (%)	ICBT-TG[VC]^b^, n/N (%)	Chi-square (*df*)	*P* value	OR^c^ (95% CI)	ICBT-SC[VC], n/N (%)	ICBT-TG[VC], n/N (%)	Chi-square (*df*)	*P* value	OR (95% CI)
**12 wk**											
	Per protocol	22/41 (54)	37/56 (66)	1.2 (1)	.22	0.59 (0.26-1.36)	19/41 (46)	34/56 (61)	1.4 (1)	.16	0.56 (0.28-1.26)
	ITT^d^	22/66 (33)	37/71 (52)	2.2 (1)	.03	0.46 (0.23-0.92)	19/66 (29)	34/71 (48)	2.3 (1)	.02	0.44 (0.27-0.89)
**9 mo**											
	Per protocol	26/38 (68)	45/51 (88)	2.2 (1)	.03	0.29 (0.10-0.86)	23/38 (61)	42/51 (82)	2.3 (1)	.03	0.33 (0.12-0.87)
	ITT	27/66 (41)	49/71 (69)	3.3 (1)	.001	0.31 (0.15-0.63)	24/66 (36)	45/71 (63)	3.1 (1)	.002	0.33 (0.17-0.66)

^a^ICBT-SC[VC]: stepped care internet-delivered cognitive behavioral therapy with step-up component delivered with therapist guidance via videoconferencing.

^b^ICBT-TG[VC]: internet-delivered cognitive behavioral therapy with full therapist delivery by videoconferencing.

^c^OR: odds ratio.

^d^ITT: intention to treat.

### Satisfaction With Treatment

Total satisfaction was rated as moderate to high at all time points for both conditions, and although scores were slightly higher for ICBT-TG[VC] than for ICBT-SC[VC] at each time point, these effects were not significantly different after 3 sessions (mean 17.42, SD 4.78 vs mean 19.02, SD 4.41 t_111_=–1.84; *P*=.07), 6 sessions (mean 18.62, SD 4.85 vs mean 20.38, SD 3.98t_96_=–1.97; *P*=.05), or 9 sessions (mean 20.59, SD 4.32 vs mean 22.25; SD 3.03; t_76_=–1.98; *P*=.05).

## Discussion

### Principal Findings

This study examined whether a stepped care ICBT model for child and adolescent anxiety was noninferior to a fully therapist-guided ICBT program when therapist guidance was delivered via videoconferencing. Noninferiority of the stepped care model could not be determined, and the results did not support the hypothesis for primary (CSR) and all secondary (parent and child anxiety reports) outcome measures at 12 weeks and all measures but the Child Anxiety Life Interference Scale (life interference) at 9 months. Thus, although the traditional tests for evaluating outcomes across treatment conditions indicated no significant differences in outcome for the continuous measures, we cannot conclude that ICBT-SC[VC] was noninferior to ICBT-TG[VC].

However, the diagnostic interview data did suggest that ICBT-SC[VC] was less effective than ICBT-TG[VC], with a significant difference in clinical gains made by participants in the 2 conditions at the 9-month assessment. Given the variation in which participants complete ICBT sessions, 9 months is considered the primary end point for this study and our previous trial of the BRAVE Online stepped care program [11]. Study completers in the ICBT-SC[VC] condition showed a remission rate for the *primary* anxiety disorder of 68% (26/38), significantly lower than the 88% (45/51) observed in the ICBT-TG[VC] condition. Remission rates for *any* anxiety disorder were also significantly lower for ICBT-SC[VC] participants (23/38, 60%) than for ICBT-TG[VC] participants (42/51, 82%) at 9 months. These rates were lower (and significantly different) in the ITT sample, with 41% (27/66) of the ICBT-SC[VC] participants free of their *primary* anxiety diagnosis and 36% (24/66) free of *any* anxiety disorder compared to 69% (49/71) and 63% (45/71) for the ICBT-TG[VC] condition, respectively. Therefore, in terms of clinical benefit, the ICBT-SC[VC] model in this study was less effective than the ICBT-TG[VC] model in which therapist guidance was provided for all sessions.

One issue of concern was that therapy compliance was lower in the ICBT-SC[VC] group than in the ICBT-TG[VC] group, with ICBT-SC[VC] participants completing fewer treatment sessions at each time point and showing a higher proportion of dropouts. However, there are several potential reasons for this, including that participants may have understood the material and were working independently with treatment skills or that they improved enough and chose not to continue. Participants receiving the therapist-guided model showed slightly higher satisfaction ratings than those receiving the stepped care model, although the results were not significantly different.

### Benefits of Stepped Care ICBT and Videoconferencing

There are several important points to note regarding the utility of stepped care ICBT using videoconferencing in the step-up phase. First, it should be noted that the changes in anxiety symptoms and social functioning assessed through parent and youth questionnaire data showed strong improvements for both therapy conditions although noninferiority could not be demonstrated. The overall benefits of the ICBT-SC[VC] treatment were comparable to improvements observed in other clinical trials. A meta-analysis of CBT and ICBT interventions for child and adolescent anxiety showed an average remission rate for *primary* anxiety diagnosis of 49.4% in the ITT sample and 56.4% in the completer sample, and for *any* anxiety diagnosis, 46.8% in the ITT sample and 52.9% in the completer sample [44]. Meta-analyses specific to adolescent anxiety reveal lower success rates, with only 36% of the ITT sample and 37% of the completer sample free of primary anxiety diagnosis after treatment [45]. Thus, the stepped care ICBT intervention delivered in this study to both children and adolescents produced good treatment outcomes compared to other F2F and ICBT treatments delivered with therapist guidance. They were also comparable, although not as strong, as those observed in our recent study [11], They were also comparable, although not as strong, as those observed in our recent study [11], where 77% of the completers receiving the same stepped care model with email therapist guidance were free of their primary anxiety diagnosis. Thus, while the ICBT-SC[VC] model was less effective than ICBT-TG[VC] in terms of clinical diagnoses, it produced benefits that suggest that it would be an appropriate treatment model for a significant proportion of children and adolescents with a primary anxiety disorder.

Second, it is worth noting that the ICBT-SC[VC] model assessed in this study, as well as the stepped care model examined in the study by March et al [11], produced session completion rates lower than the therapist-guided model of the BRAVE Online program across all previous studies. The average number of sessions completed by the ICBT-SC[VC] participants at 12 weeks was 5.61 (SD 3.03) for this study and 5.37 for the previous stepped care model [11] compared to 7.72 (SD 3.06) sessions for the ICBT-TG[VC] participants in this study and an average of 7.5 sessions in previous trials of the BRAVE Online therapist-guided (email) program [5,24]. Session completion rates were also lower for the stepped care models at 9 months (this study: mean 7.39, SD 3.44 sessions; March et al [11]: mean 6.69 sessions) compared to therapist-guided models (this study: mean 8.73, SD 3.08 sessions; March et al [24]: mean 8.66 sessions; Spence et al [5]: mean 8.20 sessions). Thus, while the stepped care model tested here may be able to produce clinical benefits, it demonstrates a lower level of therapy engagement compared to ICBT that is delivered with minimal but regular therapist guidance throughout the program, suggesting that SC may not be suitable for those young people struggling to motivate themselves or who are not interested in ICBT programs. However, the stepped care session completion rates are greater than those observed when the BRAVE Online program is delivered in a totally self-directed manner, with no therapist guidance [9]. These findings are consistent with meta-analyses demonstrating greater treatment engagement in ICBT when therapist guidance is offered [7] and also show that engagement increases with higher dosage of therapist guidance.

Third, it is interesting to note that there was a lower proportion of young people who were stepped up who went on to become free of their primary anxiety diagnosis (9/22, 41% at 12 wk; 11/20, 55% at 9 mo) compared to ICBT-SC[VC] participants who were not stepped up (13/19, 68% at 12 wk; 15/18, 83% at 9 mo). This suggests that when young people respond well to the self-directed ICBT program at step 1, they can go on and achieve excellent remission rates, similar to the ICBT-TG[VC] condition. In this study, 40% (21/53) of the young people did not require stepping up and were able to continue with self-guided sessions themselves, which, in clinical services, could represent a large saving in therapist time. However, young people who do not respond at step 1 may face ongoing difficulty progressing through treatment and achieving clinical benefits, despite stepping up to therapist guidance. It is possible that in this study, those young people who did not respond to step 1 (32/53, 60%) were those who might not have responded well to any intervention or who required additional or alternative support from the beginning of ICBT treatment. As more investigations of stepped care ICBT are undertaken, an examination of the predictors of outcomes will be essential to determine which young people may not respond to stepped care or need alternative approaches.

Finally, this study tested the efficacy of an ICBT program (BRAVE Online) that has previously only been examined with the use of email or messaging modality in the provision of the therapist-guided component. In this study, all therapist guidance in both stepped care and therapist guidance conditions was provided in synchronous, videoconferencing format. The clinical benefits of videoconferencing in supplementing the ICBT program were clear. When delivered in the ICBT-TG[VC] format, the BRAVE Online program produced remission of primary anxiety disorder for 88% (45/51) of the participants, considerably higher than the average remission rates across previous trials of the BRAVE Online program (76.5% [24]; 78%, [5]) in which therapist guidance was provided via asynchronous messaging. These remission rates were also higher than those reported in previous meta-analyses [44] and highlight the potential value of ICBT blended with therapist guidance provided via videoconferencing. Compared to email-based therapist guidance, videoconferencing requires families to commit to a scheduled time each week to meet with the therapist. This may encourage young people to complete sessions on time in preparation for the videoconferencing session, allows enhanced tailoring of the treatment to the young person’s needs, and may provide greater rapport and support when working through the program; for example, therapists in this study were able to redirect misunderstandings and demonstrate techniques by sharing their computer screen to illustrate the components of the ICBT program and apply them to the young person. This may be particularly valuable for young people with multiple anxieties or those struggling to understand CBT concepts. However, videoconferencing sessions are less flexible than asynchronous contact and may more require therapist time.

### Study Limitations

This study is the first to examine a stepped care adaptive ICBT intervention that varied the level of videoconferencing therapist guidance provided. Importantly, it involved a sample clinically diagnosed as anxious demonstrating levels of severity and comorbidity consistent with other studies reported previously, multiple informants, and psychometrically valid measures. However, the study is not without limitations. First, although it would have been beneficial to include both child and parent diagnostic interviews, clinical diagnoses were based only on a clinical interview with the young person to minimize participant burden and focus on the recipient of treatment. For children aged <12 years, parents were invited to either be present during the interview or be consulted at its conclusion. Furthermore, although assessors were blinded to group allocation, and every effort was made to maintain this blinding, including awareness of study design and hypotheses, it is possible that they may have become aware of study conditions during follow-up interviews with participants, and this is a limitation of the study. Although the study was broadly representative and included almost a third (34/118, 28.8%) of participants residing in areas outside major cities, approximately 69.7% (92/132) of the sample were in the top 2 quartiles regarding socioeconomic status, thus limiting the generalizability of results to populations with lower socioeconomic status. The findings may also not be representative of populations in other countries or cultural and sociodemographic groups; this issue could be examined with future research. It is also important to note that participants were selected from a sample of young people who had already registered for a online CBT program and therefore were interested in online care. It is possible that the findings of the study relating to adherence and outcomes may not generalize to other young people who are less amenable to online care. Participants in this study did not have common comorbidities such as severe mood disorders, autism, substance use, behavioral disorders, and suicidal ideation, and this may also limit the generalizability of the findings. Although participation in other interventions for treating their anxiety was an exclusion criterion, it is possible that participants commenced other interventions during the study period, and this is a limitation of this study.

We note that, as with most noninferiority trials, the study was limited to 2 active treatments. In the absence of a waitlist control or nonspecific control condition, the possibility that the treatment effects for both conditions do not simply reflect spontaneous remission cannot be excluded. Mitigating against this possibility are the findings from other studies showing that therapist-guided ICBT interventions are significantly more effective than no treatment [5,24], with similar results for clinic-delivered, F2F therapy. It is also important to note that the ICBT-TG[VC] program used in this study was taken to represent evidence-based ICBT, although it was amended from previously tested versions of the program. This study assumed that the therapist guidance element of our ICBT program previously delivered via email would be effective when delivered via videoconferencing, in line with other research [19-21], although this is a potential limitation of this research. A further potential limitation of this study relates to the criterion used for the margin of noninferiority that was based on our previous trials of ICBT-TG. The noninferiority margin was calculated for the primary outcome measure (clinician diagnostic severity) and extended to other outcome measures. Future research could establish individual margins of noninferiority for different outcome measures.

The dropout rates of almost 32% (21/66) in the ICBT-SC[VC] condition and 15% (11/71) in the ICBT-TG[VC] condition limited the power of the study, although such rates are not unusual in self-help web-based interventions [46]. Furthermore, it is also possible that this study miscalculated the potential effect of the intervention when setting the noninferiority margin or that the power might have resulted in a type II error, which might have contributed to the null result. The step-up criteria used and the time point at which responder status was determined may have also influenced the findings. It is possible that an earlier assessment would be more beneficial in determining the need to adapt or step up the intervention. Furthermore, the clinical nature of the assessment meant that clinician time and judgment were required to complement questionnaire scores. This limits the potential scalability of such interventions, and future research should consider ways in which artificial intelligence and risk algorithms could assist in this process. Importantly, the time investments of the therapists and the subsequent costs of ICBT-SC[VC] were not quantified here, with a full examination of the cost-effectiveness of the ICBT-SC[VC] and ICBT-TG[VC] interventions to be examined in a separate study.

Although as indicated in our trial registration, our initial intention was to base responder status on anxiety measure scores only, pilot work highlighted the need for additional clinical and contextual consideration to provide a meaningful indication of improvement, and the results may have been different if responder status were based on scores alone. In addition, we note an inconsistency in assessment terminology originally described in the trial registration, which noted our 12-week assessment as “posttreatment” and primary end point, when the intention was to follow our previous trial methodology [11], using baseline, 12-week assessment, and 9-month follow-up, with 9-month follow-up being the primary end point. Finally, although adverse effects were assessed via repeated monitoring of anxiety symptomatology, this study did not systematically assess broader adverse events throughout this trial.

### Conclusions

This noninferiority RCT found that noninferiority of the stepped care approach could not be determined. Although ICBT-SC[VC] was acceptable to families, participants completed fewer treatment sessions. Participants receiving therapist-guided ICBT with support delivered via videoconferencing demonstrated remission rates as high as those of existing models and thus present a potentially viable new treatment model that should be compared to other treatments in future research. The findings of this research have important implications for service delivery, suggesting that the lower-intensity ICBT-SC[VC] model may offer a suitable treatment model for some, especially those who respond to step 1 and are able to continue with a fully self-guided approach. Together, the stepped-care and therapist-guided models of ICBT may offer strategies for reducing long waiting lists in primary care contexts or where there are insufficient numbers of clinically trained professionals to reach all those in need, without compromising care.

## Data Availability

The datasets generated and analyzed during this study are not publicly available due to limits to participant consent, but a deidentified dataset is stored on a University of Southern Queensland data server and is available from the corresponding author on reasonable request.

## Appendix

Multimedia Appendix 1 CONSORT-eHEALTH checklist (V 1.6.1).

Multimedia Appendix 2 Proportion of participants completing each session.

Multimedia Appendix 3 Noninferiority analysis and difference in rate of change between conditions over time.

Multimedia Appendix 4 Fixed effects from the hierarchical linear model and estimated marginal means.

## Abbreviations

ADIS-C: Anxiety Disorders Interview Schedule for Children–Child version
CALIS-C: Child Anxiety Life Interference Scale–Child report
CALIS-P: Child Anxiety Life Interference Scale–Parent report
CAS-8: Children’s Anxiety Scale-8
CBT: cognitive behavioral therapy
CGAS: Children’s Global Assessment Scale
CONSORT: Consolidated Standards of Reporting Trials
CONSORT-EHEALTH: Consolidated Standards of Reporting Trials of Electronic and Mobile Health Applications and Online Telehealth
CSR: clinician severity rating
F2F: face-to-face
HLM: hierarchical linear model
ICBT: internet-delivered cognitive behavioral therapy
ICBT-SC: stepped care internet-delivered cognitive behavioral therapy
ICBT-SC[VC]: stepped care internet-delivered cognitive behavioral therapy with step-up component delivered with therapist guidance via videoconferencing
ICBT-SG: self-guided internet-delivered cognitive behavioral therapy
ICBT-TG: therapist-guided internet-delivered cognitive behavioral therapy
ICBT-TG[VC]: internet-delivered cognitive behavioral therapy with full therapist delivery by videoconferencing
ITT: intention-to-treat
OR: odds ratio
RCT: randomized controlled trial
SCAS-C: Spence Children’s Anxiety Scale–Child version
SCAS-P: Spence Children’s Anxiety Scale–Parent version
