# Supplementary figures and images for: Integrating Videoconferencing Therapist Guidance Into Stepped Care Internet-Delivered Cognitive Behavioral Therapy for Child and Adolescent Anxiety: Noninferiority Randomized Controlled Trial
Source: JMIR Ment Health. 2025 Jan 22;12:e57405. doi: 10.2196/57405 (PMC11799812; doi:10.2196/57405)

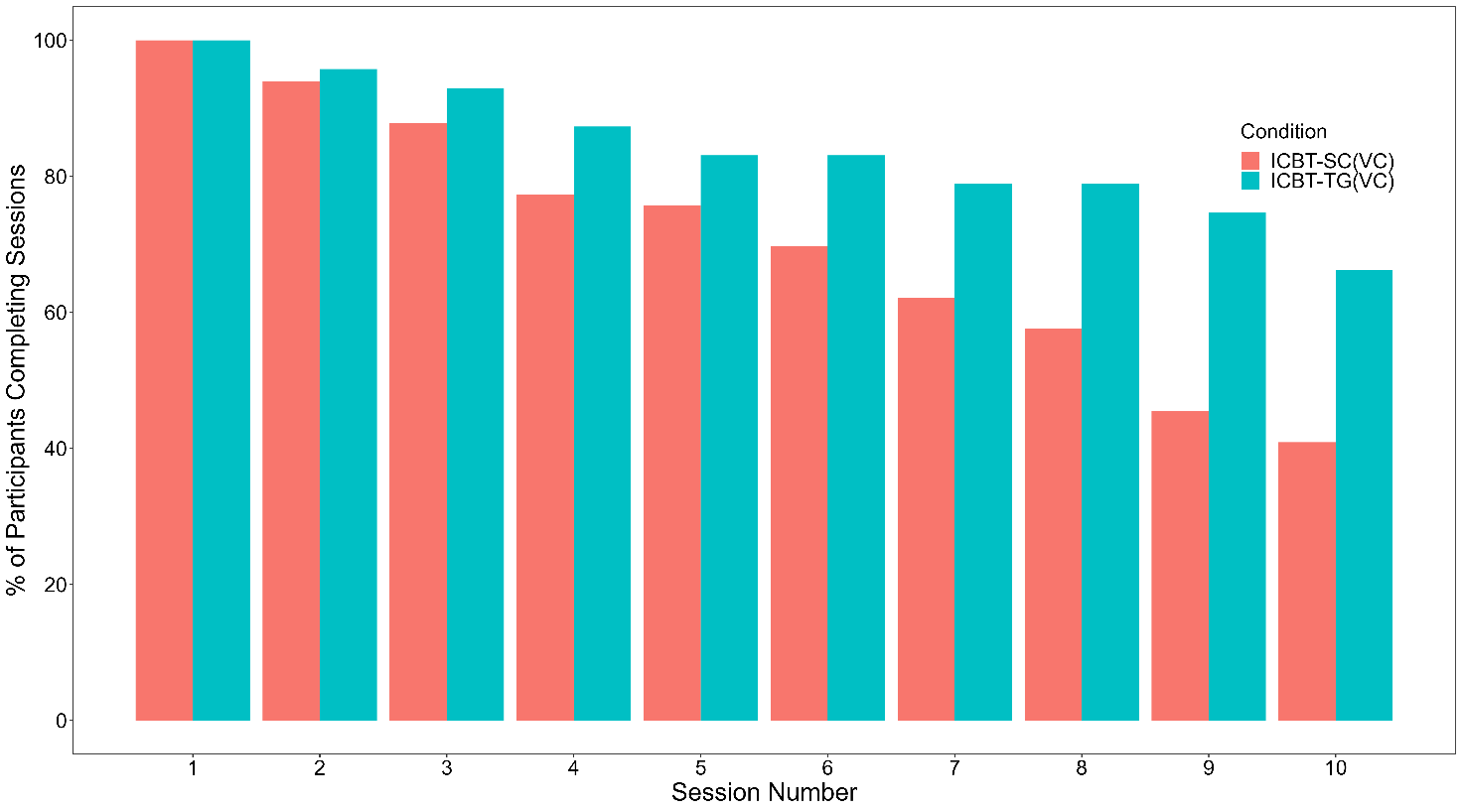


**Multimedia Appendix 2**

*Proportion of participants completing each session*

Supplement: Multimedia Appendix 2 [file mental_v12i1e57405_app2.docx]
