# Supplementary material for: Integrating Videoconferencing Therapist Guidance Into Stepped Care Internet-Delivered Cognitive Behavioral Therapy for Child and Adolescent Anxiety: Noninferiority Randomized Controlled Trial
Source: JMIR Ment Health. 2025 Jan 22;12:e57405. doi: 10.2196/57405 (PMC11799812; doi:10.2196/57405)
Supplement: Multimedia Appendix 3 [file mental_v12i1e57405_app3.docx]

**Multimedia Appendix 3**

*Non-Inferiority Analysis and Difference in Rate of Change Between Conditions from Baseline to 12-weeks and Baseline to 9-months^,^*

|  |  | Baseline to 12-weeks *^a ,b^* | | | | |  | Baseline to 9-months | | | | |
| --- | --- | --- | --- | --- | --- | --- | --- | --- | --- | --- | --- | --- |
| Scale *^c^* | *SD*^d^ | *b* [95%CI] *^e^* | *p* | Mean  Diff ^f^ | *d* | [95%CI of *d*] |  | *b* [95%CI] *^e^* | *p* | Mean  Diff ^f^ | *d* | [95%CI of *d*] |
| CSR | 0.89 | -0.73 [-1.59, 0.14] | .104 | -0.72 | -0.44 | [-0.78, -0.10] |  | -0.87 [-1.61, -0.12] | .025 | -0.861 | -0.53 | [-0.87, -0.19] |
| CGAS | 5.07 | 4.53 [-0.73, 9.79] | .094 | 4.17 | 0.40 | [0.06, 0.74] |  | 4.08 [-0.95, 9.13] | .114 | 3.931 | 0.38 | [0.04, 0.72] |
| SCAS-C | 15.70 | -11.16 [-18.24, -4.08] | .002 | -10.95 | -0.73 | [-1.07, -0.38] |  | -2.84 [-9.79, 4.10] | .424 | -3.321 | -0.22 | [-0.56, 0.12] |
| SCAS-P | 13.73 | -3.38 [-8.25, 1.49] | .176 | -3.27 | -0.26 | [-0.60, 0.07] |  | -0.93 [-6.21, 4.35] | .731 | -0.995 | -0.08 | [-0.42, 0.26] |
| CALIS-C | 6.76 | -0.87 [-4.44, 2.69] | .633 | -0.85 | -0.12 | [-0.46, 0.21] |  | 1.31 [-2.13, 4.75] | .456 | 1.390 | 0.20 | [-0.14, 0.53] |
| CALIS-P | 11.25 | -4.73 [-9.13, -0.33] | .037 | -4.58 | -0.41 | [-0.75, -0.07] |  | -1.55 [-6.15, 3.05] | .510 | -1.192 | -0.11 | [-0.44, 0.23] |

^a^ The non-inferiority margin was set to *d* = -0.40 [if the lower bound of the 95% confidence interval of the effect size does not exceed *d* = -0.40, ICBT-SC(VC) will be deemed as non-inferior to ICBT-TG(VC)], with the exception of CGAS for which higher scores indicate greater functioning with non-inferiority margin = +0.4.

^b^ All estimates of effect size based on estimated means from HLM analysis;

^c^ Abbreviations: CSR: Clinician Severity Rating; CGAS: Children’s Global Assessment Scale; SCAS-C: Spence Children’s Anxiety Scale – Child; SCAS-P: Spence Children’s Anxiety Scale – Parent; CALIS-C: Child Anxiety Life Interference Scale – Child report; CALIS-P: Child Anxiety Life Interference Scale – Parent report

^d^ SD = the pooled SD at baseline;

^e^ *b* = the difference in time slopes between ICBT-TG(VC) vs ICBT-SC(VC), with positive d values favouring greater improvement for ICBT-SC(VC) and negative values favoring ICBT-TG(VC);

^f^ Mean Diff = the difference between ICBT-SC(VC) vs ICBT-TG(VC) in change in mean scores
