# Supplementary material for: Integrating Videoconferencing Therapist Guidance Into Stepped Care Internet-Delivered Cognitive Behavioral Therapy for Child and Adolescent Anxiety: Noninferiority Randomized Controlled Trial
Source: JMIR Ment Health. 2025 Jan 22;12:e57405. doi: 10.2196/57405 (PMC11799812; doi:10.2196/57405)
Supplement: Multimedia Appendix 4 [file mental_v12i1e57405_app4.docx]

**Multimedia Appendix 4**

Fixed Effects from the HLM Analysis through baseline, 12-weeks, and 9-months

| Scale^a^ | *Effect*^b^ | *df* | *F*-value | *p* |
| --- | --- | --- | --- | --- |
| CSR | Intercept | (1, 182) | 4634.02 | < .001 |
|  | Time | (2, 182) | 269.47 | < .001 |
|  | Treatment | (1, 135) | 0.18 | .677 |
|  | Time*Treatment | (2, 182) | 2.98 | .054 |
| CGAS | Intercept | (1, 183) | 16176.53 | < .001 |
|  | Time | (2, 183) | 186.87 | < .001 |
|  | Treatment | (1, 135) | 0.24 | .628 |
|  | Time*Treatment | (2, 183) | 1.72 | .183 |
| SCAS-C | Intercept | (1, 180) | 1213.96 | <.001 |
|  | Time | (2, 180) | 124.82 | < .001 |
|  | Treatment | (1, 135) | 1.23 | .270 |
|  | Time*Treatment | (2, 180) | 7.49 | .001 |
| SCAS-P | Intercept | (1, 179) | 867.10 | <. 001 |
|  | Time | (2, 179) | 93.21 | < .001 |
|  | Treatment | (1, 134) | 1.09 | .298 |
|  | Time*Treatment | (2, 179) | 1.21 | .300 |
| CALIS-C | Intercept | (1, 181) | 852.67 | < .001 |
|  | Time | (2, 181) | 40.23 | < .001 |
|  | Treatment | (1, 135) | 0.10 | .748 |
|  | Time*Treatment | (2, 181) | 0.95 | .390 |
| CALIS-P | Intercept | (1, 180) | 733.38 | <.001 |
|  | Time | (2, 180) | 70.43 | < .001 |
|  | Treatment | (1, 134) | 0.52 | .473 |
|  | Time*Treatment | (2, 180) | 2.28 | .106 |

^a^ Abbreviations: CSR: Clinician Severity Rating; CGAS: Children’s Global Assessment Scale; SCAS-C: Spence Children’s Anxiety Scale – Child; SCAS-P: Spence Children’s Anxiety Scale – Parent; CALIS-C: Child Anxiety Life Interference Scale – Child report; CALIS-P: Child Anxiety Life Interference Scale – Parent report

^b^ All HLM models were estimated with an unstructured variance covariance matrix.

*Estimated Marginal Means and Standard Errors (SEs) for Each and Between Condition Effects for Continuous Outcome Variables at Each Assessment Occasion*

|  |  | ICBT-SC(VC) | |  | ICBT-TG(VC) | | |  | | |  |
| --- | --- | --- | --- | --- | --- | --- | --- | --- | --- | --- | --- |
| Measure*^a^* | Time point | Mean | SE |  | Mean | | SE | *d* [95% CI] *^b^*^c^ | | *p* | |
| CSR | Baseline | 5.42 | 0.20 |  | 5.42 | | 0.19 | 0.00 [-0.33, 0.34] | | .98 | |
|  | 12-weeks | 2.87 | 0.25 |  | 2.14 | | 0.22 | 0.81 [-0.19, 1.82] | | .11 | |
|  | 9-months | 1.90 | 0.26 |  | 1.04 | | 0.23 | 0.98 [0.11, 1.84] | | .028 | |
| CGAS | Baseline | 54.77 | 1.27 |  | 54.31 | | 1.23 | 0.09 [-0.25, 0.43] | | .60 | |
|  | 12-weeks | 67.67 | 1.57 |  | 71.38 | | 1.35 | -0.80 [-1.90, 0.30] | | .15 | |
|  | 9-months | 75.28 | 1.63 |  | 78.75 | | 1.42 | -0.72 [-1.77, 0.33] | | .18 | |
| SCAS-C | Baseline | 48.74 | 1.85 |  | 50.17 | | 1.79 | -0.09 [-0.43, 0.25] | | .60 | |
|  | 12-weeks | 30.70 | 2.25 |  | 21.18 | | 2.00 | 0.62 [0.24, 1.00] | | .002 | |
|  | 9-months | 24.26 | 2.39 |  | 22.37 | | 2.03 | 0.09 [-0.30, 0.48] | | .65 | |
| SCAS-P | Baseline | 37.37 | 1.53 |  | 36.73 | | 1.47 | 0.04 [-0.29, 0.39] | | .79 | |
|  | 12-weeks | 23.74 | 1.82 |  | 19.83 | 1.64 | | | 0.29 [-0.03, 0.62] | .08 | |
|  | 9-months | 21.41 | 1.91 |  | 19.78 | 1.67 | | | 0.11 [-0.21, 0.44] | .49 | |
| CALIS-C | Baseline | 16.42 | 0.86 |  | 15.97 | 0.83 | | | 0.07 [-0.27, 0.41] | .70 | |
|  | 12-weeks | 10.81 | 1.08 |  | 9.50 | 0.94 | | | 0.20 [-0.26, 0.65] | .40 | |
|  | 9-months | 8.26 | 1.13 |  | 9.19 | 0.96 | | | -0.13 [-0.56, 0.30] | .56 | |
| CALIS-P | Baseline | 26.65 | 1.37 |  | 29.32 | 1.31 | | | -0.24 [-0.58, 0.10] | .17 | |
|  | 12-weeks | 18.15 | 1.64 |  | 16.25 | 1.46 | | | 0.18 [-0.21, 0.58] | .36 | |
|  | 9-months | 15.21 | 1.73 |  | 16.70 | 1.50 | | | -0.10 [-0.49, 0.29] | .61 | |

^a^ Abbreviations: CSR: Clinician Severity Rating; CGAS: Children’s Global Assessment Scale; SCAS-C: Spence Children’s Anxiety Scale – Child; SCAS-P: Spence Children’s Anxiety Scale – Parent; CALIS-C: Child Anxiety Life Interference Scale – Child report; CALIS-P: Child Anxiety Life Interference Scale – Parent report

^b^ All values based on HLM analyses

^c^ *d* = Between groups effect size.
